# IL-6 Regulates Hepcidin Expression Via the BMP/SMAD Pathway by Altering BMP6, TMPRSS6 and TfR2 Expressions at Normal and Inflammatory Conditions in BV2 Microglia

**DOI:** 10.1007/s11064-021-03322-0

**Published:** 2021-04-09

**Authors:** Edit Varga, Ramóna Pap, Gergely Jánosa, Katalin Sipos, Edina Pandur

**Affiliations:** Department of Pharmaceutical Biology, Faculty of Pharmacy, University of Pécs, Rókus Str. 2, Pécs, 7624 Hungary

**Keywords:** Microglia, Inflammation, Iron metabolism, Hepcidin, STAT3, BMP/SMAD

## Abstract

The hormone hepcidin plays a central role in controlling iron homeostasis. Iron-mediated hepcidin synthesis is triggered via the BMP/SMAD pathway. At inflammation, mainly IL-6 pro-inflammatory cytokine mediates the regulation of hepcidin via the JAK/STAT signalling pathway. Microglial cells of the central nervous system are able to recognize a broad spectrum of pathogens via toll-like receptors and initiate inflammatory response. Although the regulation of hepcidin synthesis is well described in many tissues, little is known about the inflammation mediated hepcidin regulation in microglia. In this study, we investigated the pathways, which are involved in HAMP regulation in BV2 microglia due to inflammatory mediators and the possible relationships between the iron regulatory pathways. Our results showed that IL-6 produced by resting BV2 cells was crucial in maintaining the basal HAMP expression and hepcidin secretion. It was revealed that IL-6 neutralization decreased both STAT3 and SMAD1/5/9 phosphorylation suggesting that IL-6 proinflammatory cytokine is necessary to maintain SMAD1/5/9 activation. We revealed that IL-6 influences BMP6 and TMPRSS6 protein levels, moreover it modified TfR2 expression, as well. In this study, we revealed that BV2 microglia increased their hepcidin secretion upon IL-6 neutralization although the major regulatory pathways were inhibited. Based on our results it seems that both at inflammation and at normal condition the absence of IL-6 triggered HAMP transcription and hepcidin secretion via the NFκB pathway and possibly by the autocrine effect of TNFα cytokine on BV2 microglia.

**Supplementary Information:**

The online version contains supplementary material available at 10.1007/s11064-021-03322-0.

## Introduction

Microglial cells are tissue-resident macrophages of the central nervous system (CNS) which invade the brain at early stages of embryonic development. Under normal physiological conditions, microglia continuously survey their territorial environment, play an essential role in maintaining tissue homeostasis, in pruning of synapses, in removing apoptotic neurons from the developing CNS, regulating adult neurogenesis and maintaining neuronal health [1, 2].

As the unique immune cells of the CNS, microglia are the first line of defence and they directly become active amoeboid, highly mobile cells briskly to smallest pathological changes [3].The swift activation of microglia is a principal component of neuroinflammation, which is connected to the progression of neurodegenerative diseases. Moreover, the over-activation of microglia has detrimental neurotoxic consequences. Stimulated microglia release harmful substances, including pro-inflammatory cytokines (IL-6, IL-10, TNFα and IL-1β), reactive oxygen species (ROS), nitric oxide (NO), proteinases, and neurotoxins, but they can also provide tissue repair by releasing anti-inflammatory cytokines and neurotrophic factors [2, 4].

Microglia are able to recognize a broad spectrum of pathogens via the pattern recognition receptor family member toll-like receptors (TLRs), which sense the highly conserved pathogen-associated molecular patterns (PAMPs). Intracellular TLRs are involved in viral recognition, whereas extracellular TLRs detect a variety of microorganisms, including bacteria, parasites and fungi [5, 6]. Among the extracellular TLRs, TLR4 is the most well described member as the receptor for the major Gram-negative bacterial endotoxin, lipopolysaccharide (LPS). In aspect of Gram-positive infection, lipoteichoic acid (LTA) is the major cell wall component, which activates TLR2 [7, 8]. Both LPS and LTA can initiate the myeloid differentiation factor 88 (MyD88) operated pathway, which induces the activation of the NFκB transcription factor, the mitogen-associated protein kinase (MAPK) signalling cascade and the activator protein-1 (AP-1) to initiate the expression of pro-inflammatory genes. Furthermore, TLR4 can utilize a MyD88 independent pathway to activate NFκB and interferon regulatory factors to produce type I interferons [9–11]. In response to LPS and LTA, the activated microglial cells release interleukin-6 (IL-6) besides other pro-inflammatory cytokines, leading to generation of an inflammatory response in the CNS [12–14].

Iron is an essential element for living cells with its fundamental roles of diverse cellular processes, both in health and disease. Likewise, iron is also a key component in immune functions because of its intervention with cell-mediated immune effector pathways and cytokine activities. Inflammatory stimuli are related to important alterations in iron homeostasis, for instance pro-inflammatory cytokines are able to modify the expression of iron transport, regulatory and storage proteins in various cell types [15–17].

HAMP (hepcidin antimicrobial peptide) gene encodes a precursor of hepcidin—the 84 amino acids protein preprohepcidin. Preprohepcidin is cleaved to 60-amino acid prohepcidin, which is further amino-terminally processed and gives rise to hepcidin. There are three forms of hepcidin, the 20-, 22- and the 25-amino acid peptide, the latter the major form of it [18, 19]. The maturation of preprohepcidin-hepcidin is regulated by alpha 1-antitrypsin, a serine protease inhibitor that is able to bind prohepcidin and inhibit its cleavage into mature hepcidin [20].

The hormone hepcidin plays a central role in controlling iron homeostasis. Hepcidin causes the internalization and degradation of its receptor, the only known iron exporter, ferroportin [21]. This action leads to inhibition of iron release from enterocytes, hepatocytes and macrophages [22–24]. Besides hepatocytes, hepcidin is expressed in other cell types as well, including macrophages, adipocytes, myocytes and brain cells [22, 25, 26]. The expression of HAMP is controlled by several signals, including plasma iron level, hepatic iron stores, inflammation, erythropoietic activity, and hypoxia [27].

Iron-mediated hepcidin synthesis is triggered via the bone-morphogenetic protein/SMAD (BMP/SMAD) pathway. Increase in serum or tissue iron activates the transcription of hepcidin through BMP/SMAD signalling [27]. Current evidences suggest, that transferrin receptor 2 (TfR2) and human hemochromatosis protein (HFE) are responsible for sensing extracellular transferrin-bound iron [28]. TfR2 binds both apo- and holotransferrin (Tf) molecules and monitors the iron saturation of Tf [29]. TfR2 competes with TfR1 for HFE binding. When TfR2/HFE complex is formed as the result of the increased serum iron concentration and elevated Tf saturation, it activates HAMP expression via the extracellular signal-regulated kinases/mitogen activated phosphorylase kinase (ERK/MAPK) signalling pathway [30] and triggers intracellular iron storage and decreases iron uptake from the intestines [31]. The HFE/TfR2 pathway is involved in the signalling through the BMP/SMAD pathway to increase HAMP expression [32]. BMP6 is the predominant BMP ligand, belonging to the transforming growth factor-β (TGF-β) superfamily, which is responsible for hepcidin regulation. The minor hepcidin elevation upon chronic iron loading of BMP6 knock-out mice suggests that BMP6 pathway does not completely account for the iron response of hepcidin [27]. It was recently proposed that in the absence of TfR2, because of inadequate BMP6 synthesis low hepcidin levels were observed at increased transferrin saturation. Another key element of proper working of the BMP/SMAD pathway is the membrane-bound hemojuvelin (mHJV), a BMP co-receptor [23, 24]. Matriptase-2 [transmembrane protease serine 6 (TMPRSS6)] functions as the negative regulator of the pathway via a direct interaction and proteolytic cleavage of mHJV, resulting in increased level of soluble HJV, a suppressor of HAMP expression [23, 33].

At inflammation, mainly IL-6 cytokine mediates the regulation of hepcidin via the JAK/STAT signalling pathway. Binding of IL-6 to its receptor inchoates the phosphorylation and translocation of STAT3 to the nucleus, where it induces the upregulation of hepcidin gene. Recent studies suggest that there is a crosstalk between the inflammatory and iron-mediated signalling in hepatocytes, in which the BMP/SMAD and TfR2/HFE mediated pathways also contribute to hepcidin regulation by inflammation, however the exact mechanisms remain to be investigated [17, 34, 35].

The regulation of hepcidin synthesis is well studied in the liver, particularly in hepatocytes. Little is known about the inflammation mediated hepcidin regulation in microglia, the resident innate immune cells of the CNS. In our study, we worked with the raf/myc-immortalised murine neonatal microglia cell line BV2, which is the most frequently used substitute for primary microglia and have very similar gene expression profiling after activation [36]. While LPS is a widely used activator of microglia cells in vitro, we know a little about Gram-positive bacterial cell wall component mediated inflammatory response. In our previous study, we revealed that BV2 cells seemed to be less sensitive to LTA than LPS, but they still were able to alter the neuronal response to these inflammatory agents [37]. The mechanisms by which hepcidin is regulated in response to inflammatory stimuli generated by the presence of LPS and LTA are not fully understood.

In this study, we investigated the pathways, which are involved in HAMP regulation in microglia due to inflammatory mediators and the possible relationships between inflammatory and other iron regulatory pathways.

## Materials and Methods

### Cell Culture and Treatments

BV2 microglial cells (kindly provided by Prof. László Tretter and his research group) were cultured in Dulbecco’s Modified Eagle’s Medium (DMEM) (Lonza Ltd., Basel, Switzerland) supplemented with 10% fetal bovine serum (FBS, EuroClone S.p.A, Pero, Italy) and 1% penicillin–streptomycin (P/S, Lonza Ltd.). The cells were plated onto poly-L-ornithine (Sigma-Aldrich Kft., Budapest, Hungary) coated dishes (Sarstedt Kft., Budapest, Hungary). The experiments were carried out in a humidified atmosphere containing 5% CO_2_ at 37 °C. Concentrations and time durations of LPS and LTA treatments were selected according to the concentration dependence analyses. BV2 cells were seeded into 6-well plates and cultured for 24 h before the treatments. The cells were treated with 1 μg/mL LPS (*E.coli 055:B5*, Sigma-Aldrich Kft.) or 50 μg/mL LTA (*Staphylococcus aureus*, Sigma-Aldrich Kft.) for 9 h and 24 h in all experiments. Untreated cells were used as controls.

### Cell Viability Assay

BV2 cells were seeded into 96-well plates at a density of 10^4^ cell/well and cultured for 24 h. After LPS and LTA ± IL-6 neutralizing antibody treatments, cell viability was determined using the CCK-8 Cell Viability Kit (Sigma-Aldrich Kft.) according to the manufacturer’s protocol. Briefly, 10 µL of WST-8 reagent was added to each well, and then the plates were incubated for 1 h at 37 °C and 5% CO_2_. The reaction was stopped by adding 10 µL of 1% SDS solution to the cells. OD values of the samples were measured at 450 nm using MultiSkanGO Microplate Reader (Thermo Fisher Scientific Inc., Waltham, MA, USA) and cell viability was calculated as percentile of the cell number of the control cells.

### Neutralization of IL-6

BV2 microglial cells were seeded into 24-well plates at a density of 5 × 10^4^ cell/well and were cultured for 24 h. IL-6 neutralization was performed simultaneously with LPS or LTA treatments using anti-IL-6 monoclonal antibody (MP5-20F3, Thermo Fisher Scientific Inc.) in 100% neutralizing dose (ND). The antibody concentration was calculated as it is described in the manufacturer’s data sheet. In brief, 0.03 µg/mL antibody was used to inhibit the biological effects of 0.01 ng/mL mouse IL-6 by 50%. For the calculation, we used the IL-6 concentrations obtained from our previous ELISA measurements of LPS and LTA treated BV2 cells. The antibody concentrations were selected according to previous concentration dependence analyses, where 100% of the secreted IL-6 was inhibited (Supplementary Tables 1 and 2). Different control groups were used in the experiments in order to study the actual effect of the neutralization. Untreated cells without neutralization were used as absolute controls and untreated cells with neutralization were used as neutralized controls in each experiment.

### RNA Isolation and Quantitative Real-Time PCR

BV2 cells were seeded into 6-well culture dishes at a density of 3 × 10^5^ cell/well and were cultured for 24 h before the LPS or LTA treatments. After the treatments, cell cultures were washed with phosphate-buffered saline (PBS, Lonza Ltd.), then were harvested with trypsinization (0.05%, Lonza Ltd.). Total RNA was isolated using the Quick RNA MiniPrep Kit (Zymo Research, Irvine, CA, USA). RNA samples were reverse transcribed to cDNA from equal amounts of total RNA using the High Capacity cDNA Reverse Transcription Kit (Thermo Fisher Scientific Inc.) according to the manufacturer’s protocol. Quantitative Real-Time PCR analysis was performed using gene specific primers in a CFX96 Real-Time PCR Detection System (Bio-Rad Laboratories, Hercules, CA, USA) using iTaq Universal SYBR Green Supermix (Bio-Rad Laboratories). Data were analysed with the CFX Maestro Software (Bio-Rad Laboratories) using the comparative 2^ΔΔ^Ct (Livak) method. The expression level of the gene of interest was compared to the level of β-actin in each sample. The mRNA expressions of the treated cells were compared to the appropriate controls (9 h or 24 h). The relative expression of controls was considered as 1. Primer sequences are described in Table 1.

**Table 1:** Real-time PCR gene primer list

Primer	Sequence 5′→3′
HAMP forward	GACATTGCGATACCAATGCAG
HAMP reverse	GCAACAGATACCACACTGGGA
β-actin forward	CTGTCGAGTCGCGTCCA
β-actin reverse	TCATCCATGGCGAACTGGTG

### Western Blot Analysis

BV2 cells were seeded into 25 cm^2^ dishes at a density of 10^6^ cells and cultured for 24 h. After the treatments, cells were washed with PBS then were collected with trypsinization (0.05%). Pelleted cells were washed with ice cold PBS and were pelleted again in a clean microcentrifuge tube. BV2 cells were fractionated upon collection using the Subcellular Protein Fractionation Kit for Cultured Cells (Thermo Fisher Scientific Inc.). Protein contents of the fractions were measured with DC Protein Assay Kit (Bio-Rad Laboratories). The same amount of protein from each sample was loaded onto 10% or 12% polyacrylamide gels and separated by electrophoresis (SDS-PAGE), then transferred by electroblotting onto nitrocellulose membranes (Pall AG, Basel, Switzerland). The membranes were probed with rabbit polyclonal antibodies produced against TfR2 (1:1000; Invitrogen, Thermo Fisher Scientific Inc.), pSTAT3 (1:2000; Cell Signaling Technology Europe, Leiden, The Netherlands), pSMAD1/5/9 (1:1000; Cell Signaling Technology Europe), NFκB/p50 (1:1000; Cell Signaling Technology Europe), NFκB/p65 (1:2000; Cell Signalling Technology Europe) and TMPRSS6 (1:1000; Sigma-Aldrich Kft.) proteins. Goat anti-rabbit (H + L) HRP was used as secondary antibody (1:3000; Bio-Rad Laboratories). β-actin (1:2000, Sigma-Aldrich Kft.) was used as housekeeping control. Each protein was analysed in three independent experiments. Optical densities of the Western blots were calculated by ImageJ software [38] and expressed as percentage of target gene/β-actin abundance.

### Enzyme-Linked Immunosorbent Assay (ELISA) Measurements

After each treatment, culture media of BV2 microglial cells were collected and stored at − 80 °C until the measurements. The secreted IL-6 content of the culture media was determined with IL-6 Mouse ELISA Kit (Thermo Fisher Scientific Inc.). The secreted mature hepcidin content of the samples was determined with Mouse Hepcidin 25 ELISA Kit (Abbexa Ltd., Cambridge, UK) and the secreted BMP6 protein concentration was determined with Mouse BMP6 ELISA Kit (Cusabio Technology LLC, Houston, TX, USA). The secreted TNFα concentration was determined by Mouse TNFα ELISA Kit (Thermo Fisher Scientific Inc.). All measurements were performed in triplicate according to the instructions of the manufacturers.

### Statistical Analysis

The data presented are representative of three independent experiments, *n* corresponds to the number of independent experiments. Real-time PCR and cell and ELISA measurements were carried out in triplicate, viability assays were performed in quadruplicate in each independent experiments. Data are presented as mean ± standard deviation (SD). Statistical analysis was performed using SPSS software (IBM Corporation, Armonk, NY, USA). Statistical significance was determined by one- or two-way ANOVA (considering the number of the examined variables) followed by Tukey’s HSD post hoc test. Statistical significance was defined as *p* value < 0.05.

## Results

### Effects of LPS and LTA Treatments on Cell Viability

**Fig. 1:**
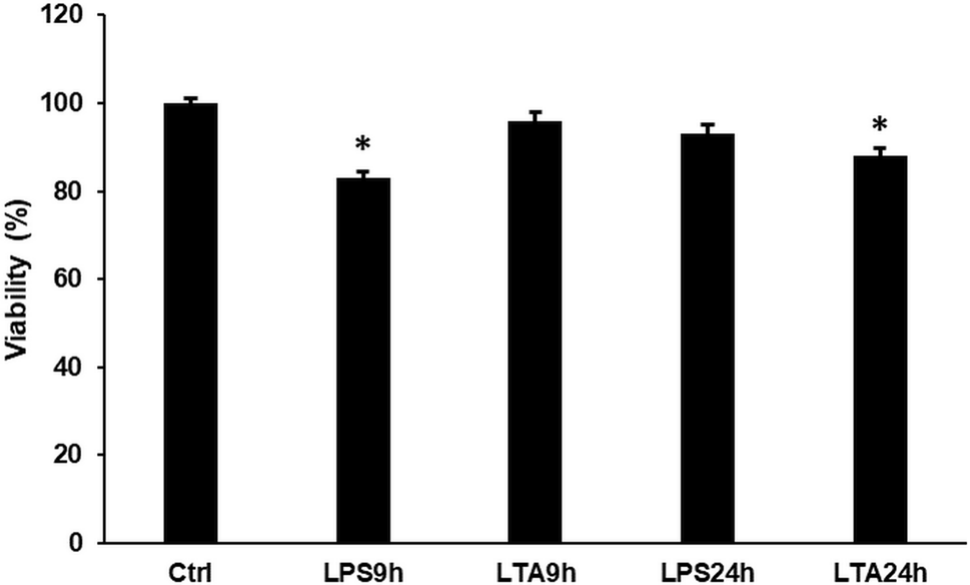
Viability measurements of LPS and LTA treated BV2 cells. Cell viability was determined using CCK-8 cell viability kit. Cell viability was calculated as percentile of the cell number of the control cells. The bars represent mean values and error bars represent standard deviation (SD) for three independent experiments (*n* = 3). The asterisk indicates *p* < 0.05 compared to the untreated control. Data was analysed by one-way ANOVA followed by Tukey’s HSD post hoc test

To investigate whether LPS or LTA exert a cytotoxic effect on BV2 cells we determined the cell viabilities upon treatments. The results showed that LPS treatment induced a statistically significant, but moderate decrease in cell viability at 9 h, while LTA treatment decreased the viability at 24 h (Fig. 1). These results suggest that LPS and LTA may exert different action on cell cycle. Therefore, we examined both time points of the treatments in the following experiments.

### Effects of LPS and LTA Treatments on IL-6 Secretion

**Fig. 2:**
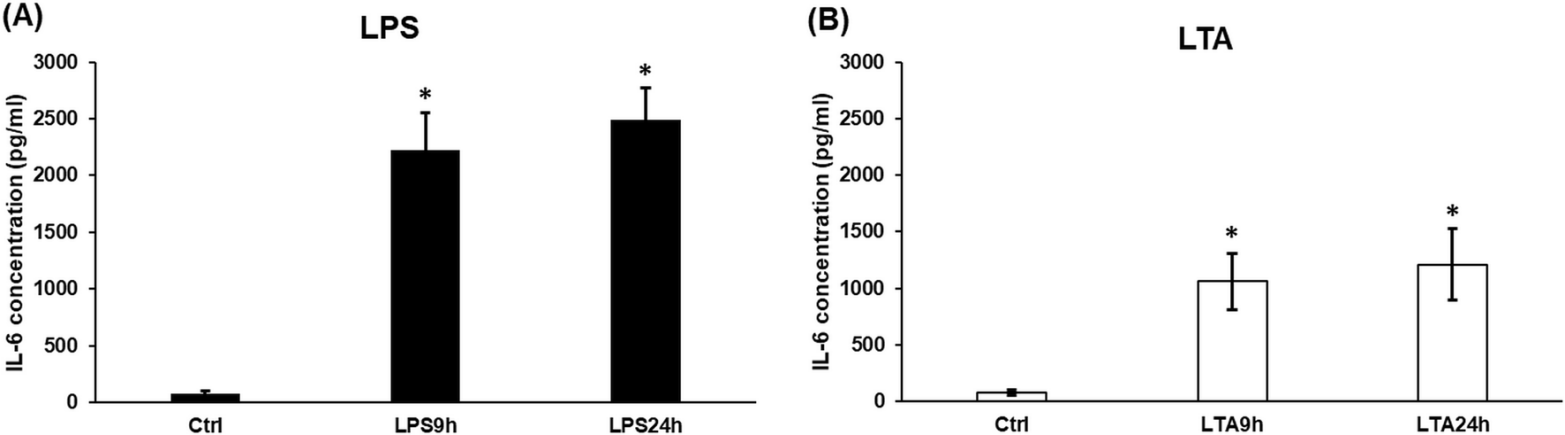
Effects of LPS and LTA on the IL-6 secretion of BV2 cells. IL-6 content of cell culture supernatants was determined with IL-6 Mouse ELISA Kit. **a** IL-6 secretion of LPS treated BV2 cells. **b** IL-6 production of LTA treated BV2 cells. The columns represent mean values and error bars represent standard deviation (SD) of three independent determinations (*n* = 3). The asterisk marks *p* < 0.05 compared to the control. Data was analysed by one-way ANOVA followed by Tukey’s HSD post hoc test

Several studies showed that the PAMPs activated microglia express and secrete remarkable amount of IL-6 [4, 39]. Therefore, we measured the concentration of the secreted IL-6 of LPS and LTA treated BV2 cells to prove that the treatments activated the microglia. We found that the cells secreted significantly increased IL-6 into the culture medium at 9 h and 24 h at both treatments, but LPS had a stronger effect on IL-6 production (Fig. 2b). Moreover, there was a consecutive increase in the IL-6 secretion with time at both treatments (Fig. 2a, b).

### Activation of the JAK/STAT3 Pathway at LPS and LTA Treatments

**Fig. 3:**
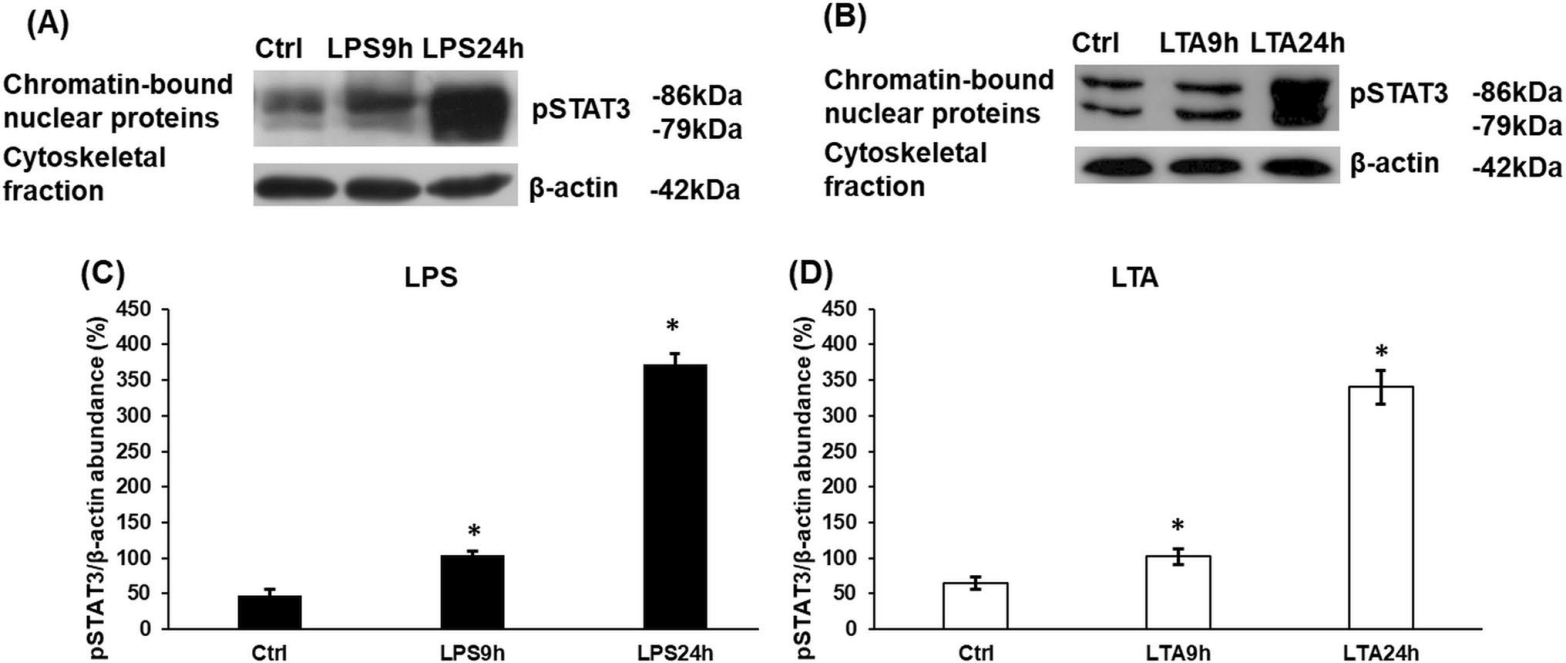
Western blot analysis of phospho-STAT3 transcription factor in LPS and LTA treated BV2 cells. BV2 cells were fractionated upon collection using the Subcellular Protein Fractionation Kit. Protein contents of the fractions were measured with DC Protein Assay Kit. The same amount of protein from each sample was loaded onto 10% polyacrylamide gels and were separated by electrophoresis, then were transferred by electroblotting onto nitrocellulose membranes. The membranes were probed with rabbit polyclonal antibodies produced against pSTAT3 according to the manufacturer’s protocol. The β-actin was used as housekeeping control. **a**, **b** Western blot analyses of pSTAT3. **c**, **d** Optical density analyses of pSTAT3. Optical densities were calculated by ImageJ software and expressed as percentage of target gene/β-actin abundance. The columns represent mean values and error bars represent standard deviation (SD) of three independent experiments (*n* = 3). The asterisk marks *p* < 0.05 compared to the control. Data was analysed by one-way ANOVA followed by Tukey’s HSD post hoc test

IL-6 pro-inflammatory cytokine is a well-known activator of the JAK/STAT3 pathway in many cell types [18, 24]. It has been proven that LPS treatment increases STAT3 phosphorylation via the activation of pro-inflammatory cytokine production or by the activation of MAPK [40]. Therefore, we investigated whether the chosen LPS and LTA concentrations were successful in increasing the phosphorylation of STAT3 transcription factor in BV2 cells. We found that LPS, as well as LTA treatment significantly increased phospho-STAT3 protein level in BV2 cells in a time dependent manner compared to the control (Fig. 3).

### Effects of LPS and LTA Treatments on Hepcidin Expression

**Fig. 4:**
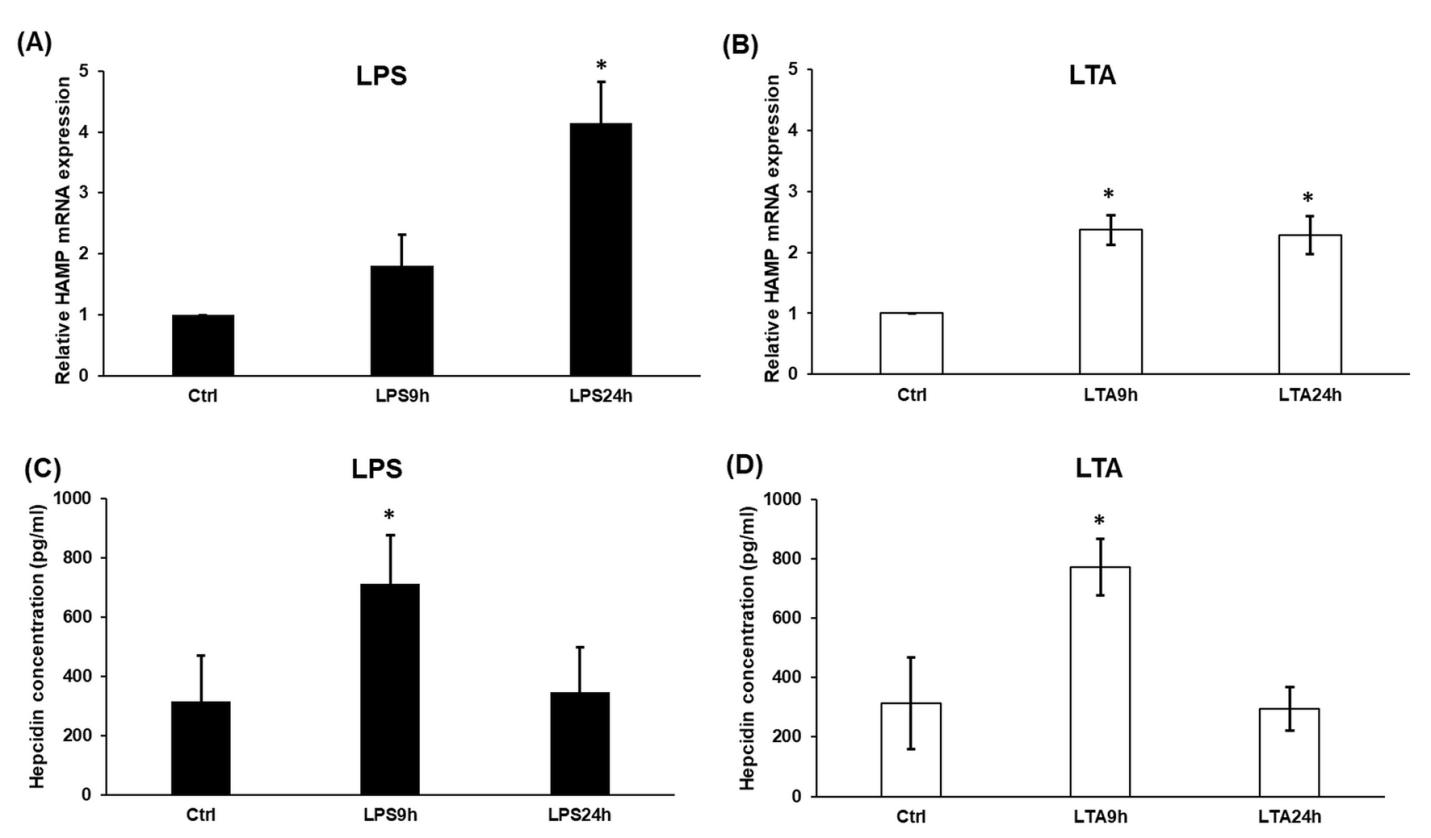
Real-time PCR analysis of HAMP and ELISA measurements of secreted hepcidin of LPS and LTA treated BV2 cells. Quantitative Real-Time PCR analysis was performed using gene specific primers and SYBR Green method. The expression level of the gene of interest was compared to the level of the β-actin in each sample. The mRNA expressions of the treated cells were compared to the appropriate untreated controls (9 h or 24 h). The relative expression of the controls was regarded as 1. Hepcidin content of cell culture supernatants was determined with Mouse hepcidin-25 ELISA Kit. **a**, **b** mRNA levels of HAMP. **c**, **d** Secreted hepcidin concentrations of LPS and LTA treated BV2 cells. The columns represent mean values and error bars represent standard deviation (SD) of three independent determinations (*n* = 3). The asterisk indicates *p* < 0.05 compared to the untreated controls. Data was analysed by two-way ANOVA followed by Tukey’s HSD post hoc test

The JAK/STAT3 signalling pathway is the strongest activator of HAMP expression at inflammation [41]. After investigated the pSTAT3 protein level of LPS and LTA treated BV2 cells, we examined HAMP expression as well as the secreted hepcidin protein level. We found that the relative HAMP mRNA expression was induced in LPS treated cells, significant change was seen after 24 h treatment (Fig. 4a). Besides, LTA treatment significantly increased HAMP mRNA expression at both time points (Fig. 4b). Interestingly, the secreted hepcidin protein level was markedly increased at both treatments at 9 h compared to the control, but after the 24 h long treatments, hepcidin concentration was decreased to the control level (Fig. 4c, d). The possible explanation for these results is that prohepcidin may not mature into hepcidin completely, but may act as a signalling protein [20].

### Changes of the STAT3 Signalling Pathway After Neutralization of IL-6

**Fig. 5:**
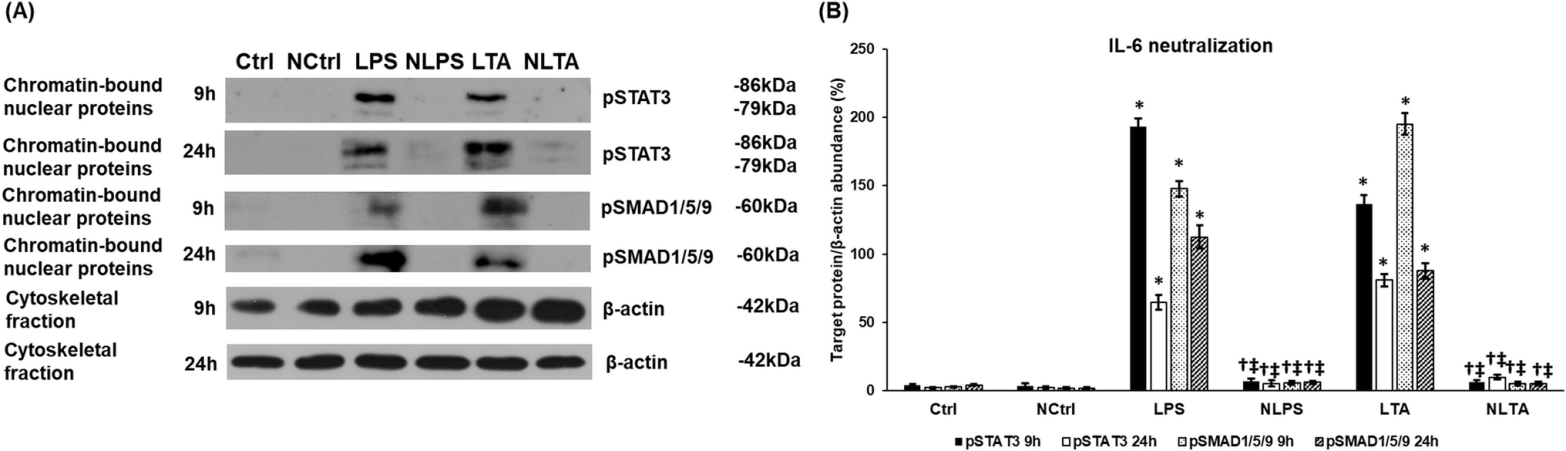
Changes of the STAT3 and SMAD1/5/9 signalling pathways after neutralization of IL-6 in LPS and LTA treated BV2 cells. The treated cells were collected and fractionated. Protein contents of the fractions were measured and the same amount of protein from each sample was loaded onto 10% polyacrylamide gels and were separated by electrophoresis, then were transferred by electroblotting onto nitrocellulose membranes. The membranes were probed with rabbit polyclonal antibodies produced against pSTAT3 or pSMAD1/5/9 according to the manufacturer’s protocols. The β-actin was used as housekeeping control. **a** Western blot analyses of pSTAT3 and pSMAD1/5/9 in the chromatin-bound nuclear fractions of LPS and LTA treated BV2 cells. **b** Optical density analyses of pSTAT3 and pSMAD1/5/9 proteins. Optical densities were calculated by ImageJ software and expressed as percentage of target gene/β-actin abundance. The columns represent mean values and error bars represent standard deviation (SD) of three independent experiments (*n* = 3). *Ctrl* untreated control, *NCtrl* control + neutralizing IL-6 antibody, *NLPS* neutralizing IL-6 antibody + LPS, *NLTA* neutralizing antibody + LTA. The asterisk indicates *p* < 0.05 compared to the control. The cross marks *p* < 0.05 compared to NCtrl. The double cross indicates significant difference, *p* < 0.05 between LPS and NLPS or LTA and NLTA. Data was analysed by two-way ANOVA followed by Tukey’s HSD post hoc test

We decided to reveal whether the pSTAT3 pathway induced by LPS and LTA treatments can crosstalk with the BMP/SMAD signalling pathway in regulating HAMP expression. For the investigation, we neutralized the secreted IL-6 using anti-IL-6 antibody treatment both on control and LPS or LTA treated BV2 cells. Neutralization of IL-6 did not affect cell viability (Supplementary Fig. 1). The efficiency of different concentrations of neutralizing antibody on IL-6 secretion can be seen in Supplementary Tables 1 and 2. We tested if the neutralization of IL-6 suppressed the phosphorylation of STAT3. Western blot analysis showed that the chromatin-bound pSTAT3 protein level drastically decreased in neutralized cells compared to LPS and LTA treated cells at each examined time points (Fig. 5). These results prove that secreted IL-6 acts via autocrine way causing the phosphorylation of STAT3 transcription factor. Next, we examined the phosphorylated SMAD1/5/9 protein level to reveal whether there was a possible crosstalk with the JAK/STAT pathway. The results showed that pSMAD1/5/9 level decreased parallel with the pSTAT3 level suggesting that IL-6 was necessary for maintaining the activity both STAT3 and SMAD pathways (Fig. 5).

### Effects of IL-6 Neutralization on Hepcidin Secretion

**Fig. 6:**
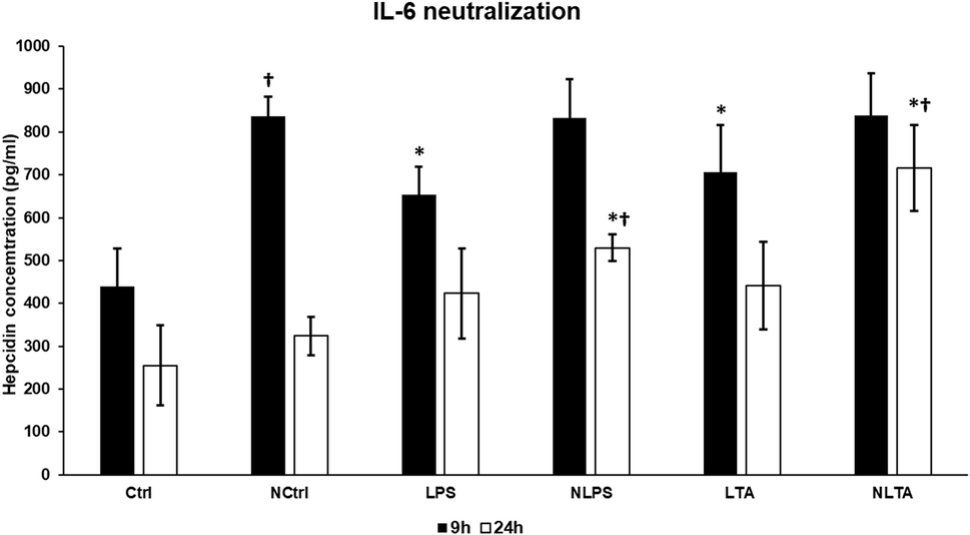
ELISA measurement of secreted hepcidin of IL-6 neutralized, LPS or LTA treated BV2 cells. Hepcidin content of cell culture supernatants was determined with Mouse hepcidin-25 ELISA Kit. The columns represent mean values and error bars represent standard deviation (SD) of three independent measurements (*n* = 3). *Ctrl* untreated control, *NCtrl* control + neutralizing IL-6 antibody, *NLPS* neutralizing IL-6 antibody + LPS, *NLTA* neutralizing antibody + LTA. The asterisk indicates *p* < 0.05 compared to the controls. The cross marks the significant difference, *p* < 0.05 between neutralized and non-neutralized samples. Data was analysed by two-way ANOVA followed by Tukey’s HSD post hoc test

Since both STAT3 and SMAD act as positive regulators of HAMP expression [23, 24], we studied the effect of IL-6 neutralization on hepcidin secretion. Interestingly, the secreted hepcidin protein level increased after neutralization at 9 h compared to the LPS and LTA treatments (Fig. 6). Significant hepcidin elevation was also found after the neutralization of the control cells. Moreover, at 24 h significant hepcidin elevation was found in the supernatants of the neutralized LPS and LTA treated cells, and of the neutralized control, as well (Fig. 6). It was revealed that neutralization of the LTA treated cells exerted a stronger effect on hepcidin secretion at 24 h compared to the LPS neutralization (Fig. 6). These results propose that not only the JAK/STAT3 pathway but BMP/SMAD pathway are important in regulating hepcidin production at inflammation, but other signalling pathways can take their roles contributing hepcidin secretion. The results also support our hypothesis that an interaction occurs between the JAK/STAT3 and other hepcidin regulatory pathways.

### Effects of IL-6 Neutralization on BMP6 Expression

**Fig. 7:**
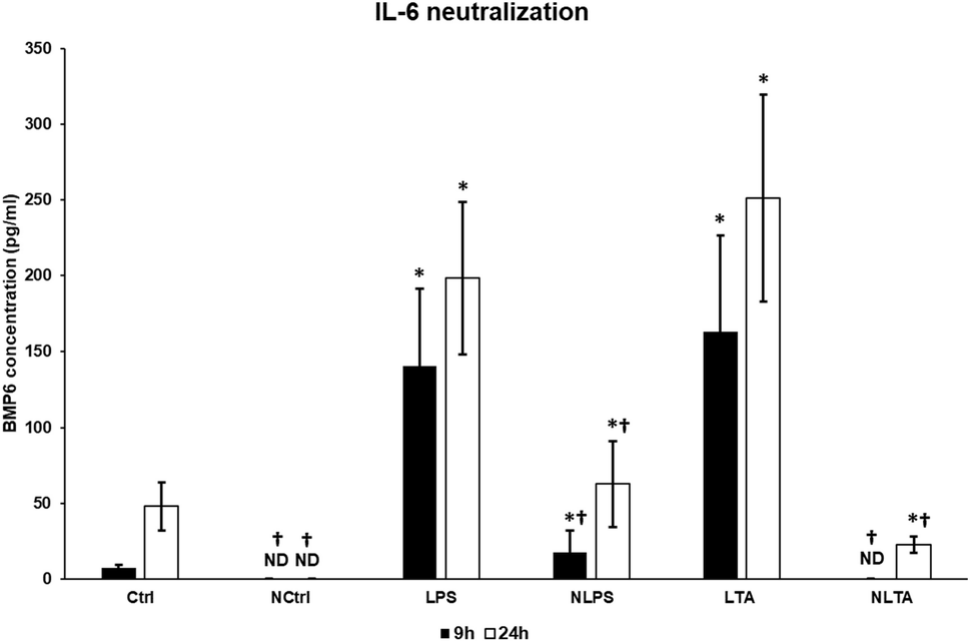
ELISA measurement of secreted BMP6 of IL-6 neutralized and LPS or LTA treated BV2 cells. BMP6 content of cell culture supernatants was determined with Mouse BMP6 ELISA Kit according to the instructions of the manufacturer. The columns represent mean values and error bars represent standard deviation (SD) of three independent measurements (*n* = 3). *Ctrl* untreated control, *NCtrl* control + neutralizing IL-6 antibody, *NLPS* neutralizing IL-6 antibody + LPS, *NLTA* neutralizing antibody + LTA. The asterisk indicates *p* < 0.05 compared to the controls. The cross marks the significant difference, *p* < 0.05 between neutralized and non-neutralized samples. Data was analysed by two-way ANOVA followed by Tukey’s HSD post hoc test

To assess which signalling molecule of the BMP/SMAD pathway is affected by IL-6 cytokine, we measured the concentration of BMP6, the ligand of the BMP receptor [24], in the culture medium. BMP6 secretion strongly decreased in control cells after IL-6 neutralization (Fig. 7). This reduction was detected in neutralized LPS and LTA treated cells, as well. Moreover, in the case of LTA, blocking of IL-6 drastically reduced BMP6 secretion after 9 h treatment (Fig. 7). Based on our results it is supposed that the BMP6 expression is regulated by IL-6 mediated pathway, and the downregulation of BMP6 contributes to the decreasing pSMAD level observed in the neutralized cells.

### Effects of LPS and LTA Treatments and IL-6 Neutralization on TMPRSS6 and TfR2 Expressions

**Fig. 8:**
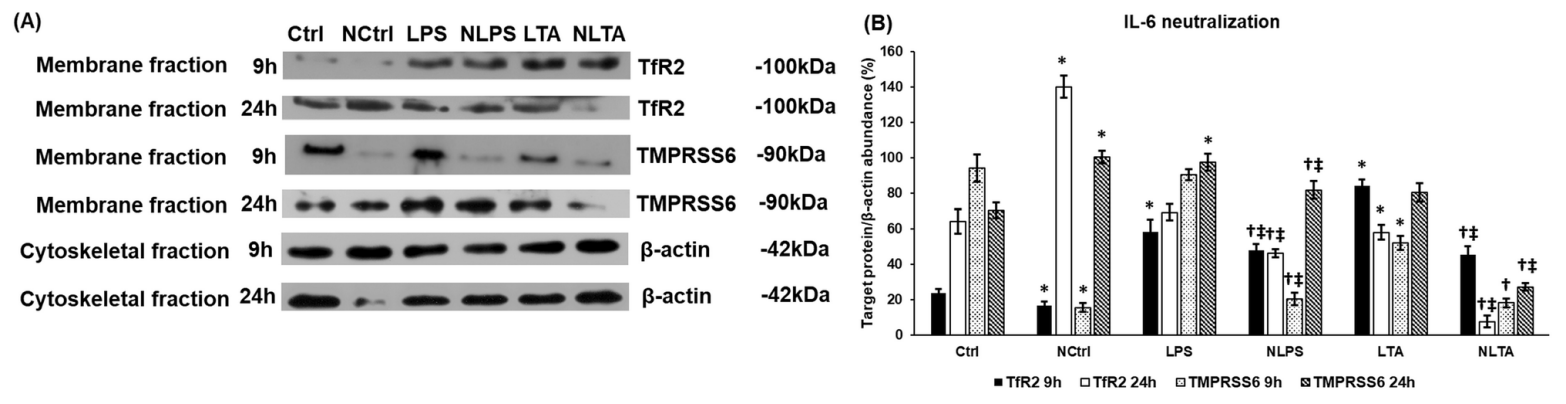
Western blot analysis of TfR2 and TMPRSS6 protein levels in IL-6 neutralized and LPS or LTA treated BV2 cells. BV2 cells were fractionated upon collection using the Subcellular Protein Fractionation Kit. Protein contents of the fractions were measured and the same amount of protein from each sample was loaded onto 10% polyacrylamide gels and were separated by electrophoresis, then were transferred by electroblotting onto nitrocellulose membranes. The membranes were probed with rabbit polyclonal antibodies produced against TfR2 and TMPRSS6 according to the manufacturer’s protocols. β-actin was used as housekeeping control. **a** Western blot analyses of TfR2 and TMPRSS6. **b** Optical density analyses of TfR2 and TMPRSS6. Optical densities were calculated by ImageJ software and expressed as percentage of target gene/β-actin abundance. The columns represent mean values and error bars represent standard deviation (SD) of three independent experiments (*n* = 3). *Ctrl* untreated control, *NCtrl* control + neutralizing IL-6 antibody, *NLPS* neutralizing IL-6 antibody + LPS, *NLTA* neutralizing antibody + LTA. The asterisk marks *p* < 0.05 compared to Ctrl. The cross marks *p* < 0.05 compared to NCtrl. The double cross indicates significant difference, *p* < 0.05 between LPS and NLPS or LTA and NLTA. Data was analysed by two-way ANOVA followed by Tukey’s HSD post hoc test

We examined also the protein expression of TMPRSS6 or matriptase-2, which is a negative regulator of HAMP expression [24, 42]. Stimulation of BV2 cells with LPS did not affect TMPRSS6 protein level at 9 h but elevated it at 24 h; meanwhile LTA treatment decreased its level at 9 h. At 24 h, LTA caused slight elevation of TMPRSS6 compared to the control (Fig. 8a, b). Inhibition of the JAK/STAT3 pathway by IL-6 neutralization further decreased the protein level of TMPRSS6 but both at 9 h and 24 h compared to the LPS or LTA treated cells (Fig. 8a, b). Based on the results it is supposed that TMPRSS6 downregulation could be able to contribute to HAMP expression even though the BMP6 production of BV2 cells decreased.

Next, we turned to another positive HAMP regulatory protein, TfR2 that can trigger HAMP expression via the ERK/MAPK pathway [30]. We found that the protein level of TfR2 increased significantly at 9 h in LPS treated cells compared to the control (Fig. 8a, b). LTA treatment increased TfR2 protein level at 9 h but decreased it at 24 h (Fig. 8a, b). These results suggest that TfR2 may help to increase HAMP expression at the early stage of the treatments. Blocking the JAK/STAT3 pathway by IL-6 neutralization significantly decreased TfR2 in both treatments at each time points suggesting that the JAK/STAT3 pathway interacts with TfR2 regulated pathway and the activity of STAT3 influences the expression of TfR2 (Fig. 8a, b).

It is interesting to note that neutralization of the control cells also led to decreasing TfR2 and TMPRSS6 protein levels at 9 h suggesting that the IL-6 activated signalling pathway is necessary to maintain basal HAMP expression.

### Effect of IL-6 Neutralization on NFκB/p50 and NFκB/p65 Expressions

**Fig. 9:**
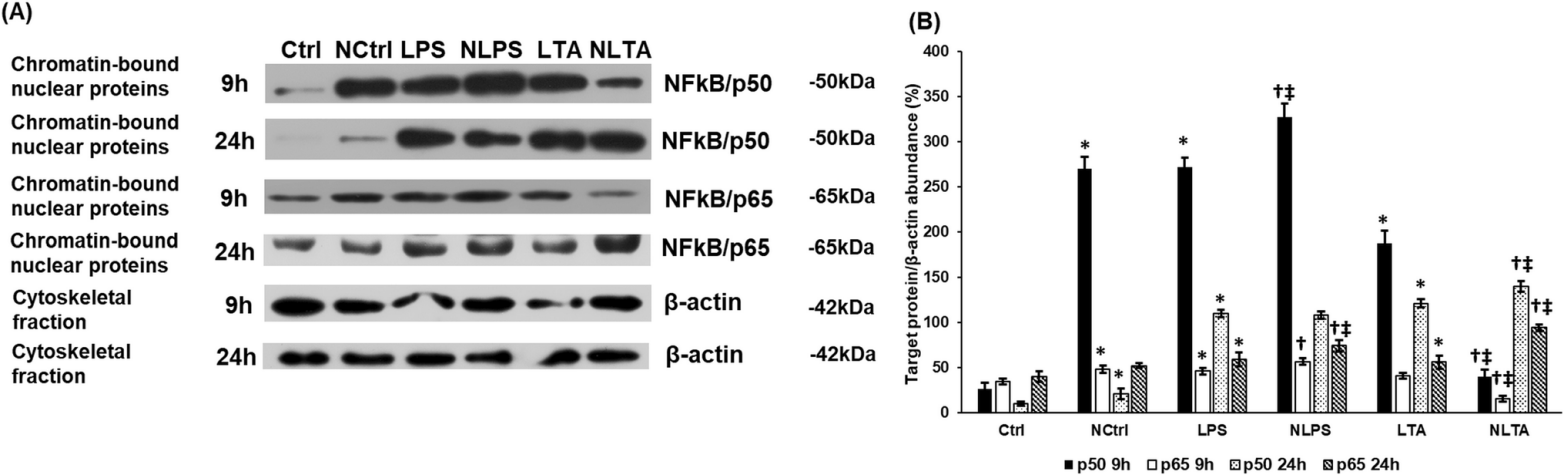
Changes of the NFκB signalling pathway after neutralization of IL-6 in LPS and LTA treated BV2 cells. The treated cells were collected and fractionated. Protein contents of the fractions were measured and the same amount of protein from each sample was loaded and separated onto 12% polyacrylamide gels, then were transferred by electroblotting onto nitrocellulose membranes. The membranes were probed with rabbit polyclonal antibodies produced against p50 and p65 according to the manufacturer’s protocols. The β-actin was used as housekeeping control. **a** Western blot analysis of p50 and p65 in the chromatin-bound nuclear fractions of LPS and LTA treated BV2 cells. **b** Optical density analysis of p50 and p65 proteins. Optical densities were calculated by ImageJ software and expressed as percentage of target gene/β-actin abundance. The columns represent mean values and error bars represent standard deviation (SD) of three independent experiments (*n* = 3). *Ctrl* untreated control, *NCtrl* control + neutralizing IL-6 antibody, *NLPS* neutralizing IL-6 antibody + LPS, *NLTA* neutralizing antibody + LTA. The asterisk marks *p* < 0.05 compared to Ctrl. The cross marks *p* < 0.05 compared to NCtrl. The double cross indicates significant difference, *p* < 0.05 between LPS and NLPS or LTA and NLTA. Data was analysed by two-way ANOVA followed by Tukey’s HSD post hoc test

The above data show that none of the examined pathways that positively regulate HAMP expression work properly after IL-6 neutralization. Therefore, we further studied NFκB signalling pathway that is also able to induce HAMP expression [43, 44] and like STAT3, is activated by bacterial cell wall components [37]. Only phosphorylated p50 and p65 are able to bind to DNA and activate transcription, we used the chromatin bound protein fraction in the Western blot experiments to determine NFκB activation [45, 46]. Both LPS and LTA treatments increased p50 level at 9 h compared to the control, meanwhile p65 level increased at 9 h after LPS and did not change significantly after LTA treatments (Fig. 9a and b). Blocking of the JAK/STAT3 pathway increased both p50 and p65 levels in case of LPS treatment, but decreased them in case of LTA treated cells (Fig. 9a, b). At 24 h, both LPS and LTA increased p50 and p65 protein levels, meanwhile neutralization of IL-6 exerted positive effect on LTA treated cells but only increased p65 level in LPS treated cells (Fig. 9a, b). These results show that both LPS and LTA treatments and IL-6 neutralization triggered different effects on BV2 cells. We need to note that in control cells, neutralization induced both NFκB/p50 and p65 protein expressions after 9 h and 24 h treatments, suggesting that the reduction in IL-6 concentration around the BV2 cells may activate a compensatory mechanism to restore IL-6 production. The alterations in p50 and p65 protein levels in case of neutralized LPS or LTA treated cells may be responsible for the changes in hepcidin secretion.

### TNFα Expression Did Not Change Following IL-6 Neutralization

**Fig. 10:**
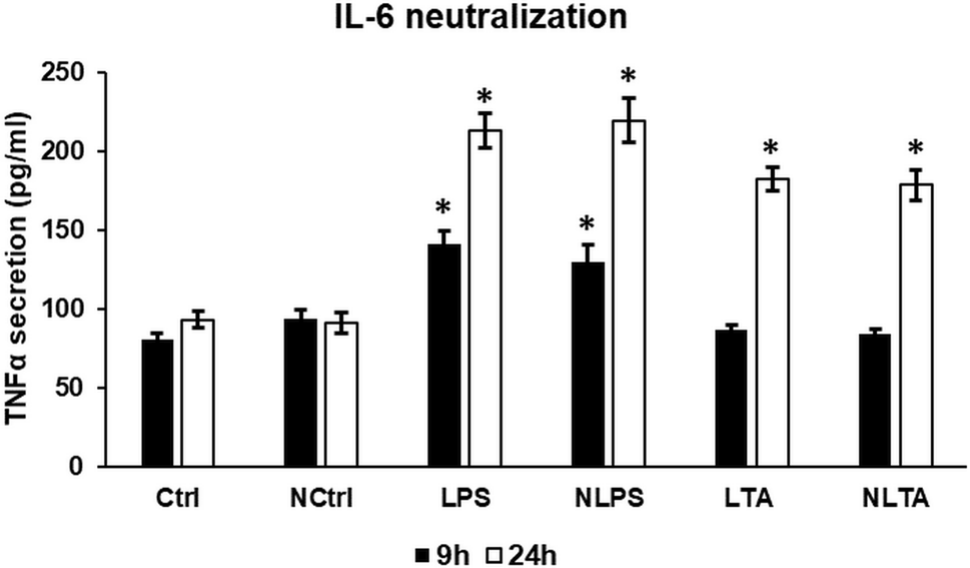
ELISA measurement of secreted TNFα of IL-6 neutralized and LPS or LTA treated BV2 cells. TNFα content of cell culture supernatants was determined with Mouse TNFα ELISA Kit according to the instructions of the manufacturer. The columns represent mean values and error bars represent standard deviation (SD) of three independent measurements (*n* = 3). *Ctrl* untreated control, *NCtrl* control + neutralizing IL-6 antibody, *NLPS* neutralizing IL-6 antibody + LPS, *NLTA* neutralizing antibody + LTA. The asterisk indicates *p* < 0.05 compared to the controls Ctrl and NCtrl, respectively. Data was analysed by two-way ANOVA followed by Tukey’s HSD post hoc test

TNFα acts as the activator of NFκB as well as HAMP expression, while the NFκB signalling pathway induces the expression of TNFα cytokine [17]. To test, if IL-6 neutralization affects the expression of TNFα, we determined the concentration of secreted TNFα after LPS and LTA treatments, and IL-6 neutralization. We found that activation of BV2 cells with LPS or LTA increased TNFα secretion (Fig. 10). Interestingly, there were no significant alterations in TNFα protein concentrations in the different treated groups after neutralization (Fig. 10). These results suggest that there is no interaction between the STAT3 pathway and TNFα secretion. The TNFα activated signalling pathways may contribute to the increased hepcidin production when the JAK/STAT3 pathway is inhibited.

## Discussion

Hepcidin is the main regulator of systemic iron homeostasis by binding to and causing the internalization and degradation of the iron exporter, ferroportin [18]. Ferroportin degradation results in iron retention in the cells, therefore hepcidin increases intracellular iron stores and decreases serum iron level [47]. Hepcidin is predominantly expressed by hepatocytes, but it is also detectable in other cell types, including microglia, the resident immune cells of the central nervous system [21, 24, 25]. Its gene, HAMP is regulated in response to several stimuli, including iron stores, transferrin saturation, erythropoietic activity, hypoxia and inflammation [22, 23]. The connections between the HAMP regulatory pathways still need further clarification, however, several studies deal with possible crosstalks [48, 49] between the JAK/STAT and the BMP/SMAD signalling pathways.

In our study, we focused on the effects of bacterial cell wall components, LPS and LTA on hepcidin regulation in BV2 cells, a commonly used microglial cell line. First, we revealed that LPS activated microglia in a 50 times lower concentration compared to LTA, suggesting that microglia react stronger with Gram-negative bacteria. The possible reason for this powerful concentration difference may be the different mechanism of action of LPS and LTA [37]. Interestingly, LPS decreased cell viability at 9 h while LTA treatment reduced it later suggesting the different effects of these cell wall components on BV2 cells. The IL-6 cytokine measurements showed that both LPS and LTA treatments increased significantly IL-6 secretion although LPS treated cells showed approximately two times higher IL-6 concentration compared to LTA treated cells.

IL-6 pro-inflammatory cytokine is one of the strongest positive regulator of HAMP mRNA expression via the activation of the JAK/STAT3 pathway [19, 22, 23]. As discussed above, LPS and LTA administration resulted in elevated IL-6 production in microglia cells, therefore we examined both HAMP mRNA expression and hepcidin secretion of treated BV2 cells. Our results showed that LPS and LTA treatments acted different ways on HMAP mRNA expression; the HAMP mRNA level was lower in case of LTA treatment compared to LPS treated cells. These results demonstrate that LPS acted slower but stronger on HAMP expression suggesting alterations in HAMP transcriptional regulation. The secreted hepcidin protein levels were also elevated, but behaved somewhat differently compared to the mRNA expression. This discrepancy may be the result of the posttranslational maturation of prohepcidin to mature hepcidin, which is regulated by several factors such as furin proprotein convertase and alpha-1 antitrypsin protease inhibitor [20, 50]. The concentration and activity of these regulators determine the maturation process of hepcidin. Moreover, prohepcidin also acts as a repressor of HAMP transcription in the nucleus of the cell [51].

The phosphorylation of STAT3 transcription factor in LPS stimulated microglia is widely proved, meanwhile increasing evidence show that LTA is also able to activate the STAT3 signalling pathway [52, 53]. We revealed that LPS and LTA treatments exerted different effect on HAMP mRNA expression, therefore we examined whether the cause of this alteration was the distinct phosphorylation rate of STAT3 transcription factor. Western blot analysis showed that phosphorylated STAT3 protein levels significantly increased in both cases in the chromatin-bound nuclear protein fraction supposing that the pathway was activated and pSTAT3 was translocated to the nucleus. Although at 24 h pSTAT3 levels increased, the HAMP mRNA expression was not elevated in case of LTA treatment. This result suggest that other HAMP regulators may interfere with the action of pSTAT3 [51].

According to the latter hypothesis, we investigated if the elimination of secreted IL-6 from the culture medium affected hepcidin secretion and whether other HAMP regulatory pathways were affected by pSTAT3 downregulation. First, we tested different concentrations of IL-6 neutralizing antibody that were calculated based on the amount of secreted IL-6 cytokine (Supplementary Table 1). To determine the effectiveness of anti-IL-6 antibody, we examined pSTAT3 protein level using Western blot. The results showed drastically decreased pSTAT3 levels in neutralized cells compared to LPS and LTA treated cells. Interestingly, the downregulation of STAT3 phosphorylation increased hepcidin secretion of BV2 cells instead of decreasing it, even in the neutralized control cells. These noteworthy results raised the possibility that there should be other factors/signalling pathways, which are involved in hepcidin expression at inflammation in microglia.

Besides inflammation, hepcidin is regulated by the body iron status. One of the important iron- mediated regulatory mechanism operates through the BMP/SMAD pathway [54]. The key endogenous ligand of the BMP/SMAD pathway is BMP6. Recent studies demonstrate that there is a possible crosstalk between the BMP/SMAD and the IL-6 mediated JAK/STAT3 pathway at inflammation in hepatoma cell lines and primary mouse hepatocytes [48, 49]. It was shown that BMP6 was effective in increasing HAMP expression only in the presence of LPS in macrophages [55]. Mice lacking SMAD4 were not able to induce HAMP expression at IL-6 treatments [56, 57]. Moreover, in Hep3B hepatoma cells, inhibition of BMP signalling diminished the IL-6 mediated hepcidin induction [58] suggesting that BMP/SMAD pathway regulates the IL-6 mediated JAK/STAT pathway.

Our results show that the inhibition of the JAK/STAT3 by IL-6 neutralization decreased the phosphorylation of SMAD1/5/9 proposing that IL-6 act directly or indirectly on the BMP/SMAD pathway. To reveal which component of the BMP signalling is regulated by IL-6 mediated pathway we examined the changes in BMP6 secretion at LPS and LTA treatments and at IL-6 neutralization. BMP6 measurements revealed significant increase in the secretion of BMP6 after both LPS and LTA treatments, indicating that inflammatory stimuli may trigger the BMP/SMAD pathway in BV2 microglia cells via upregulating the secretion of BMP6. Interestingly, BMP6 levels were significantly reduced after neutralization of IL-6 in LPS and LTA treated cells that demonstrate the key role of IL-6 in the expression of BMP6 at inflammation. Moreover, BMP6 secretion decreased in neutralized control cells as well, suggesting that the IL-6 regulated signalling pathway contributes to the basal activity of the BMP/SMAD signal transduction. BMP6 acts as a transcriptional regulator of IL-6 [59], therefore the downregulation of BMP6 by IL-6 neutralization may contribute to the further decrease in IL-6 production. The decreasing level of BMP6 leads to the reduced activity of BMPR that also works as a positive regulator of p38 MAPK. The decreased activity of p38 contributes to the downregulation of IL-6, as well [59]. Serine phosphorylation of STAT transcription factors is crucial in the activation of transcription of the target genes [60, 61]. The downregulation of p38 by the reduced BMP6 production may also implicated in the decreased activity of STAT3. On the other hand, the STAT3 phosphorylation is necessary for the interaction with SMAD transcription factor, therefore the decreased phosphorylation of STAT3 implies reduced SMAD activation [62–64].

TMPRSS6 or matriptase-2 acts as a negative regulator of HAMP transcription by cleaving mHJV to soluble form thus decreasing the activity of the BMP/SMAD pathway [65]. It is also supposed that TMPRSS6 expression is not dependent on the BMP/SMAD pathway [66]. We examined whether changes in TMPRSS6 protein level could contribute to increased hepcidin production. LPS treatment increased TMPRSS6 level, but we found significant decrease in case of LTA treatment. This alteration may be responsible for increasing HAMP expression and work together with the IL-6 activated pathway. Nevertheless, neutralization of IL-6 significantly decreased TMPRSS6 protein level both at LPS and LTA treatments, which was reciprocally proportional with the concentration measurement of secreted hepcidin. We found significant reduction in TMPRSS6 level in case of neutralized controls suggesting that not only BMP6 but TMPRSS6 expression was linked to IL-6 regulated signal transduction. One possible explanation can be the increasing intracellular iron content contributing also to the downregulation of TMPRSS6 [67]. TMPRSS6 transcription is regulated by STAT5 transcription factor, but for the proper activity of STAT5, p38 mediated serine phosphorylation is crucial [66, 68]. The downregulation of p38 by the decreasing activity of BMP/SMAD pathway may be involved in the reduced TMPRSS6 expression at IL-6 neutralization.

TfR2 acts as an iron sensor in hepatocytes [69] and interacts with HFE to maintain basal hepcidin synthesis by activating the ERK/MAPK pathway [70]. It is also supposed that TfR2 interacts with BMP/SMAD signalling in the cytoplasm [71, 72]. According to the above described function of TfR2 we hypostatized that IL-6 affects this regulatory pathway, as well. We determined the protein level of TfR2 at LPS and LTA treatments and upon IL-6 neutralization. LPS treatment increased TfR2 protein level, while LTA decreased it. This alteration may be the underlying cause of the difference found in HAMP mRNA expression of LPS and LTA treated BV2 cells. Due to neutralization of IL-6, TfR2 level decreased at both LPS and LTA treatments suggesting that TfR2 expression is dependent on the IL-6 regulated signalling pathway. The reduced level of TfR2 may lead to the decreasing activity of MAPK, which is responsible for SMAD phosphorylation. Moreover, the downregulation of ERK may contribute to the decreasing serine phosphorylation of STAT that is implicated in SMAD activation [73].

The examined three regulatory factors BMP6, TMPRSS6 and TfR2 seemed to be in contact with the IL-6/JAK/STAT pathway: inhibition of the STAT signalling by IL-6 neutralization decreased not only STAT3 phosphorylation, but also BMP6, TMPRSS6 and TfR2 expressions and influenced the activity of the BMP/SMAD pathway, as well. These interactions between the HAMP regulatory pathways unravelled that IL-6 acted as a multiregulatory protein, which affected different signalling pathways. IL-6 is very important in maintaining the normal hepcidin production of BV2 cells. Although our findings show a complex interaction network between the HAMP regulatory pathways in BV2 microglia, the elevation of hepcidin secretion due to the inhibition of STAT3 phosphorylation cannot be explained completely by these results.

Since we used bacterial cell wall components LPS and LTA as inflammatory mediators, we turned to the NFκB signalling pathway regulated by TLRs, the receptors of LPS and LTA, which is also involved in hepcidin regulation [40]. It seems that LPS and LTA acted differently on the signalling proteins of the NFκB pathway. These fluctuations may be the result of different velocity of TLR receptor turnover, by which the receptors are internalized from the cell surface then recycled into the plasma membrane [74]. IL-6 neutralization increased the levels of both p50 and p65 in case of LPS treatment but decreased them in case of LTA suggesting that IL-6 may activate a compensatory mechanism to restore IL-6 secretion, but it seemed that LPS and LTA modify differently this process.

The LPS/LTA activated NFκB pathway crosstalks with the IL-6/STAT3 pathway. McFarland et al. have been described that inhibition of STAT3 pathway leads to activation of NFκB pathway [75]. The downregulation of STAT3 phosphorylation by IL-6 neutralization of BV2 cells can contribute to the increasing activity of the NFκB pathway, which triggers hepcidin synthesis. Moreover, unphosphorylated STAT3 transcription factors can bind to NFκB subunits forming dimers, which translocate into the nucleus and activate the expression of NFκB-dependent genes [76], which may further act on hepcidin expression.

We also examined whether another NFκB and HAMP transcriptional regulator, TNFα could contribute the aforementioned results [77, 78]. We found that LPS was more efficient on TNFα secretion, although LTA exerted positive effect as well, on TNFα. Based on the results TNFα may contribute to elevated hepcidin secretion at LPS and LTA treatments of BV2 microglia via the activation of the NFκB pathway. We cannot exclude that at inflammation the changes in intracellular iron content also activate the NFκB pathway [79–82] inducing HAMP expression. Our results proved that decreasing STAT3 phosphorylation did not alter TNFα production suggesting that the JAK/STAT and TNFα signalling are independent from each other. Moreover, TNFα may further inhibit the BMP/SMAD pathway by downregulating HJV expression independently from the inflammatory pathway [17].

In our study, we induced inflammation using LPS and LTA bacterial cell wall components. We found that both components induced hepcidin secretion via the IL-6 mediated JAK/STAT pathway, although LPS exerted stronger reaction in BV2 microglia. We investigated the interactions between the JAK/STAT and the BMP/SMAD signalling pathways regulating HAMP transcription in BV2 microglia. We found that inhibition of JAK/STAT pathway via the elimination of IL-6, altered the protein levels of BMP6, TMPRSS6 and TfR2 that are directly or indirectly involved in the regulation of the BMP/SMAD pathway. The results prove that inflammation affects the BMP/SMAD pathway at distinct levels. We also proved that inhibition of the major inflammatory HAMP regulatory pathway, the NFκB pathway and the TNFα production of BV2 cells were involved in the upregulation of hepcidin secretion. Taken together, IL-6 produced by resting BV2 cells was crucial in maintaining the basal HAMP expression and hepcidin secretion by regulating the BMP/SMAD signalling network. However, the same or similar mechanisms may work in other microglia cell lines (e.g. HMC-3) or primary microglia, further investigation is needed to clarify this question.

## Supplementary Information

Below is the link to the electronic supplementary material.

Supplementary file 1 (DOCX 358 kb)

Supplementary file 2 (PDF 290 kb)

## Data Availability

All data generated or analysed during this study are included in this published article.
