# Supplementary material for: IL-6 Regulates Hepcidin Expression Via the BMP/SMAD Pathway by Altering BMP6, TMPRSS6 and TfR2 Expressions at Normal and Inflammatory Conditions in BV2 Microglia
Source: Neurochem Res. 2021 Apr 9;46(5):1224–38. doi: 10.1007/s11064-021-03322-0 (PMC8053173; doi:10.1007/s11064-021-03322-0)
Supplement: Supplementary file 1 — (DOCX 358 kb) [file 11064_2021_3322_MOESM1_ESM.docx]

**Neurochemical Research**

**IL-6 regulates hepcidin expression via the BMP/SMAD pathway by altering BMP6, TMPRSS6 and TfR2 expressions at normal and inflammatory conditions in BV2 microglia**

Edit Varga, Ramóna Pap, Gergely Jánosa, Katalin Sipos and Edina Pandur*

Department of Pharmaceutical Biology, Faculty of Pharmacy, University of Pécs, H-7624, Rókus Str. 2., Pécs, Hungary

*Corresponding author:

Edina Pandur

e-mail: [edina.pandur@aok.pte.hu](mailto:edina.pandur@aok.pte.hu)


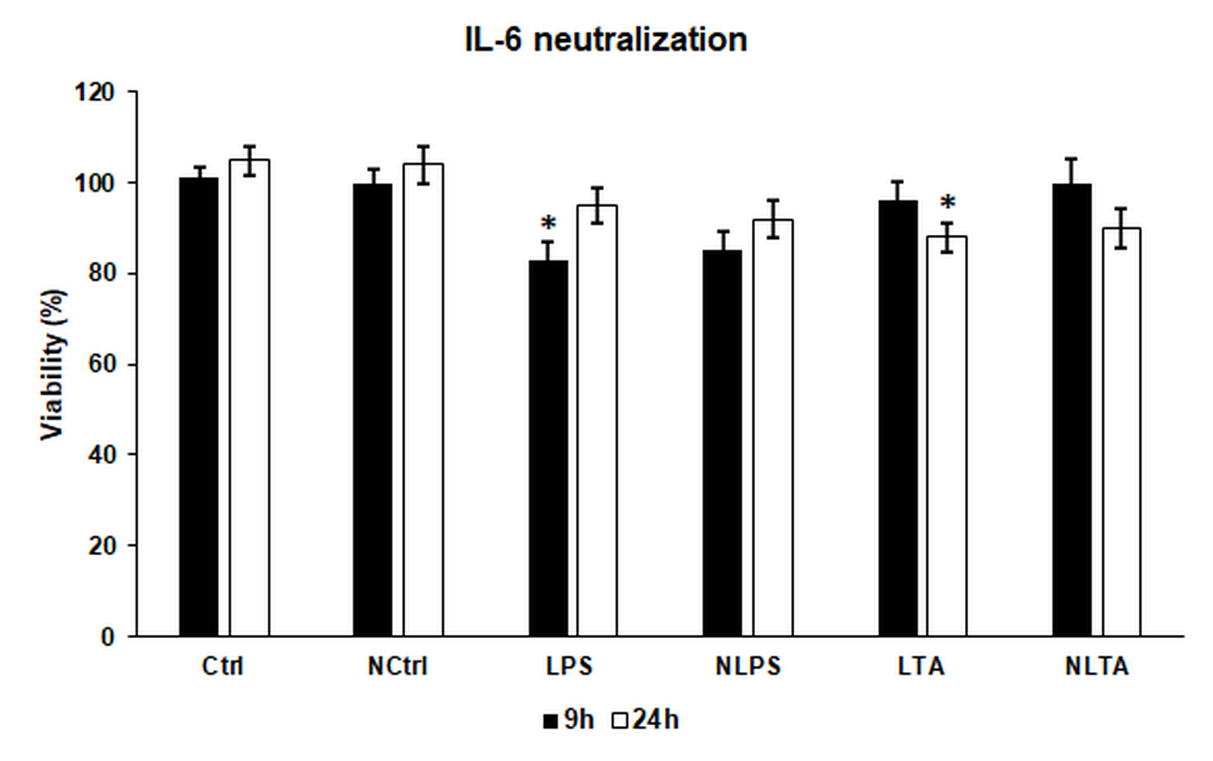


**Supplementary figure 1.** Viability of BV2 cells after 9 h and 24 h long LPS and LTA or after neutralization of IL-6 cytokine. Cell viability was determined using CCK-8 cell viability kit. Cell viability was calculated as percentile of the cell number of the control cells. Abbreviations: Ctrl-untreated control, NCtrl-control+neutralizing IL-6 antibody, NLPS-neutralizing IL-6 antibody+LPS, NLTA-neutralizing antibody+LTA. The bars represent mean values and error bars represent standard deviation (SD) for three independent experiments (*n* = 3). The asterisk indicates *p* < 0.05 compared to the untreated control. Data was analysed by one-way ANOVA followed by Tukey’s HSD post hoc test.

No significant change was revealed between Ctrl and NCtrl, LPS and NLPS, LTA and NLTA proving that IL-6 neutralization with anti-IL-6 antibody did not affect cell viability.

| **Supplementary table 1** IL-6 concentrations after IL-6 neutralization | | | | | |
| --- | --- | --- | --- | --- | --- |
| **9 h treatments** | **Mean (pg/mL)** | **SD** | **24 h treatments** | **Mean (pg/mL)** | **SD** |
| Ctrl | 89,16 | 11,42 | Ctrl | 166,85 | 4,90 |
| NCtrl | 9,92 | 1,63 | NCtrl | 11,46 | 3,81 |
| LPS | 2367,03 | 113,65 | LPS | 2655,31 | 44,90 |
| NLPS 50% | 12,23 | 0,54 | NLPS 50% | 14,54 | 2,72 |
| NLPS 75% | 4,92 | 1,09 | NLPS 75% | 17,62 | 5,98 |
| NLPS 100% | 1,85 | 1,09 | NLPS 100% | 9,15 | 3,81 |
| NLPS 120% | 2,62 | 2,18 | NLPS 120% | 8,00 | 5,44 |
| LTA | 1201,08 | 70,71 | LTA | 1199,92 | 107,49 |
| NLTA 50% | 6,46 | 2,18 | NLTA 50% | 4,87 | 0,07 |
| NLTA 75% | 9,15 | 8,16 | NLTA 75% | 2,62 | 1,09 |
| NLTA 100% | 3,38 | 2,18 | NLTA 100% | 3,69 | 1,74 |
| NLTA 120% | 3,77 | 0,54 | NLTA 120% | 4,62 | 1,52 |

| **Supplementary table 2** Concentrations of IL-6 neutralizing antibody used in the experiments | | | |
| --- | --- | --- | --- |
| **9 h treatments** | **anti-IL-6 antibody concentration (µg/mL) for 100% inhibition** | **24 h treatments** | **anti-IL-6 antibody concentration (µg/mL) for 100% inhibition** |
| NCtrl | 0,534 | NCtrl | 1,001 |
| NLPS | 14,202 | NLPS | 15,93 |
| NLTA | 7,206 | NLTA | 7,199 |

The antibody concentrations were selected according to previous concentration dependence analyses, where 100% of the secreted IL-6 was inhibited. For the 100% inhibition we used 0.06 µg/mL antibody against 0.01 ng/mL secreted IL-6.
