# Supplementary figures and images for: IL-6 Regulates Hepcidin Expression Via the BMP/SMAD Pathway by Altering BMP6, TMPRSS6 and TfR2 Expressions at Normal and Inflammatory Conditions in BV2 Microglia
Source: Neurochem Res. 2021 Apr 9;46(5):1224–38. doi: 10.1007/s11064-021-03322-0 (PMC8053173; doi:10.1007/s11064-021-03322-0)

Figure 3AB

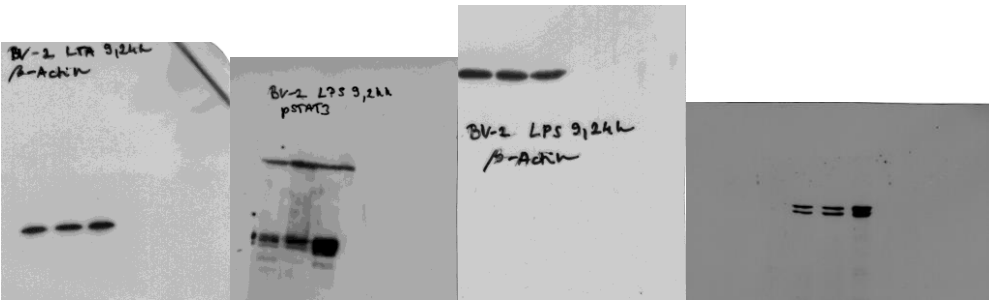

Figure 5A

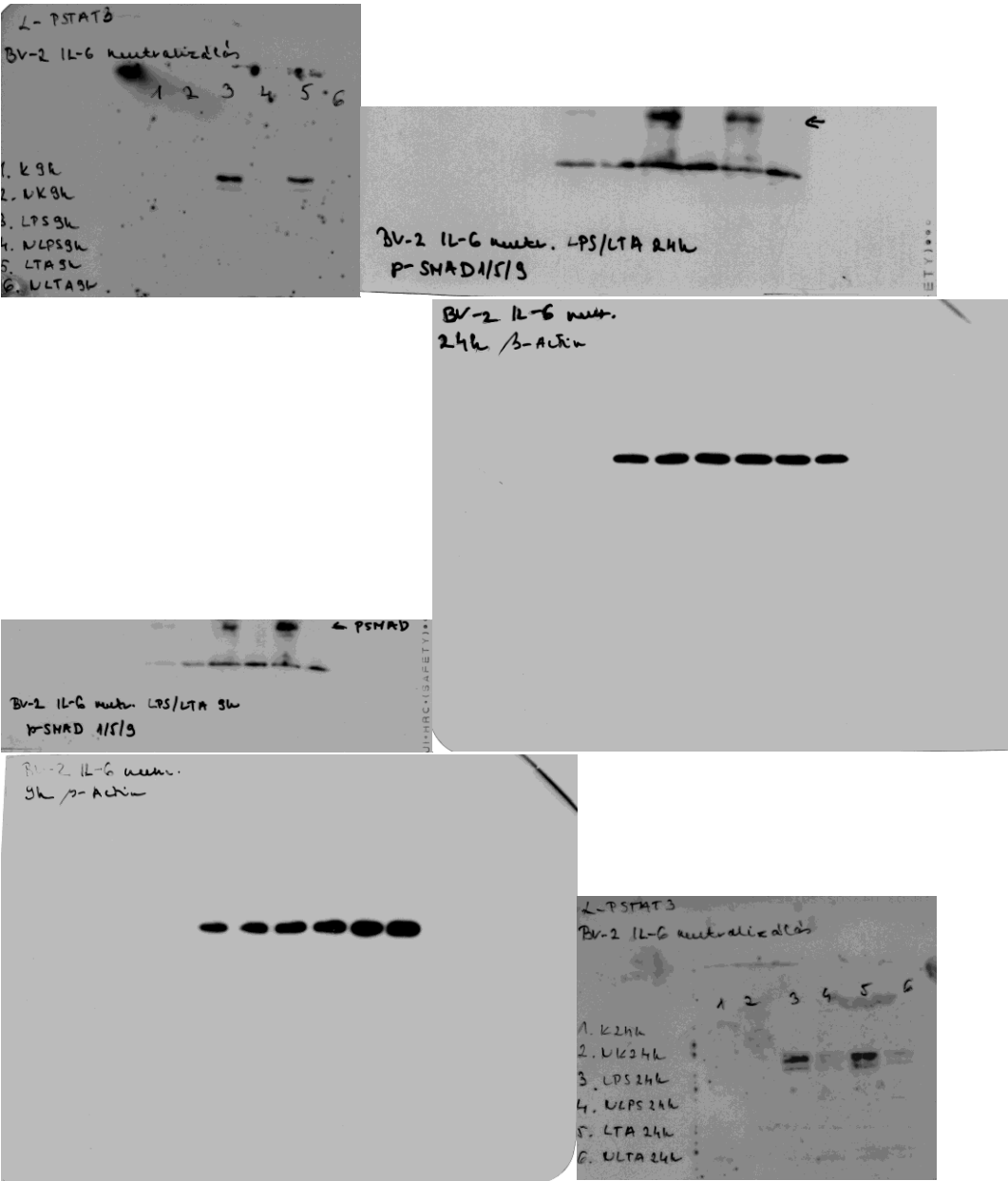

**Figure 8A**

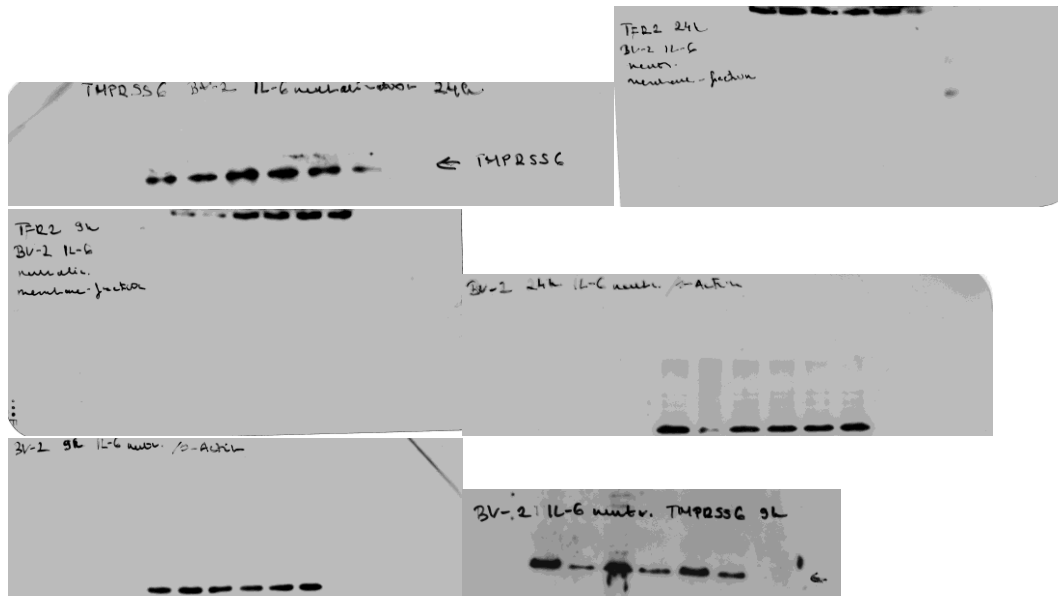

**Figure 9A**

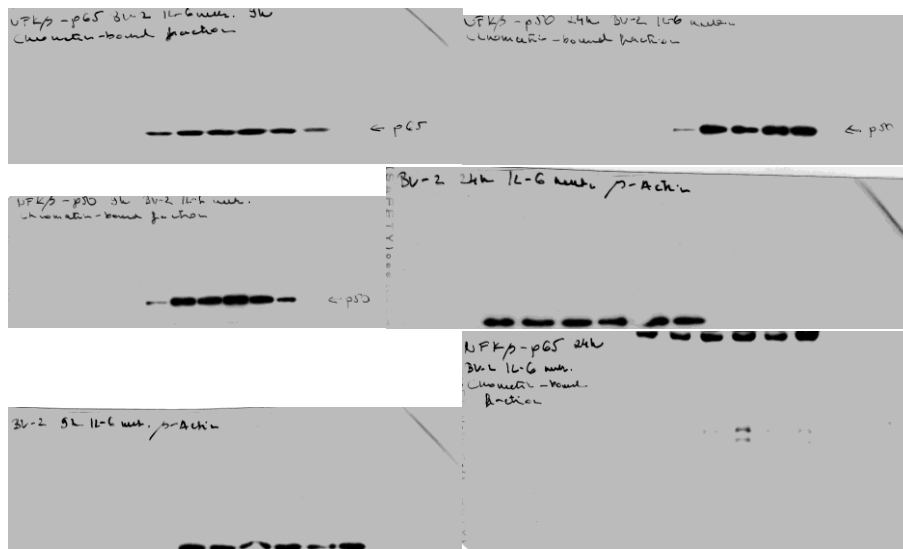

Supplement: Supplementary file 2 — (PDF 290 kb) [file 11064_2021_3322_MOESM2_ESM.pdf]
